# Clarifying the mechanisms of the light-induced color formation of apple peel under dark conditions through metabolomics and transcriptomic analyses

**DOI:** 10.3389/fpls.2022.946115

**Published:** 2022-07-28

**Authors:** Xiaomin Xue, Shoule Tian, Ru Chen, Xueping Han, Jinzheng Wang, Xianyan Zhao

**Affiliations:** ^1^Shandong Institute of Pomology, Tai’an, China; ^2^State Key Laboratory of Biobased Material and Green Papermaking, School of Bioengineering, Qilu University of Technology, Jinan, China

**Keywords:** apple, light induction, dark coloration, metabolome, transcriptome

## Abstract

Many studies have demonstrated that anthocyanin synthesis in apple peel is induced by light, but the color of bagged apple peel continues to change under dark conditions after light induction has not been characterized. Here, transcriptional and metabolic changes associated with changes in apple peel coloration in the dark after different light induction treatments were studied. Apple pericarp can achieve a normal color under complete darkness followed by light induction. Metabolomics analysis indicated that the expression levels of cyanidin-3-O-galactoside and cyanidin-3-O-glucoside were high, which might be associated with the red color development of apple peel. Transcriptome analysis revealed high expression levels of *MdUFGT*s, *MdMYB*s, and *MdNAC*s, which might play a key role in light-induced anthocyanin accumulation under dark conditions. 13 key genes related to dark coloring after light induction was screened. The results of this study provide new insights into the mechanism of anthocyanin synthesis under dark conditions.

## Introduction

Apples (*Malus domestica* Borkh.) are deciduous fruit trees in the Rosaceae family that are popular among consumers worldwide for their rich nutrient profiles, which include sugars, acids, vitamins, flavonoids, pectins, and proteins (Hecke et al., 2006; Zhang et al., 2010). Furthermore, apples provide several health benefits to the cardiovascular system (Winter et al., 2018) and liver (Zhou Y. et al., 2020) and possess cancer-fighting compounds (Wang and Stoner, 2008; Sampaio et al., 2021). Apples are widely cultivated in temperate regions and have become a major source of income for many farmers.

The color of apples is an important indicator of their quality and commercial value, especially for red apple varieties (Deng et al., 2018). The red color of apples is determined by the level of anthocyanins. Anthocyanin synthesis and its regulation have thus been the focus of much research (Espley et al., 2007; An et al., 2012; Li et al., 2021).

Light is one of the main factors affecting the synthesis of anthocyanins. In Arabidopsis and many other plants, anthocyanins can only be synthesized under light, and they do not accumulate under weak light or dark conditions (Maier and Hoecker, 2015). Strong light promotes the expression of structural genes and transcription factors, whereas weak light or dark environments inhibit the expression of genes involved in anthocyanin synthesis (Jeong et al., 2004). The expression of *MdMYB1* and *MdMYB10* is up-regulated under strong light, and this induces the expression of downstream genes and promotes the synthesis of anthocyanins in apple (Gu et al., 2019). MdTCP46 forms a protein complex with MdMYB1 under strong light conditions and promotes anthocyanin synthesis by enhancing the ability of MdMYB1 to activate the expression of downstream genes (An et al., 2020). In apple, decreases in anthocyanin synthesis under dark conditions are related to the interaction between MdCOP1 and MdMYB1, and ubiquitination degrades MdMYB1. Under light conditions, MdCOP1 is transported out of the nucleus, and the degradation of MdMYB1 ceases, which stabilizes the function of MdMYB1 and results in the synthesis of anthocyanins (Li et al., 2012; Kang et al., 2021). Previous studies have shown that the anthocyanins of apple peel do not accumulate in dark environments.

In this study, we found that apple peel could synthesize anthocyanins under dark conditions following light induction, which is in contrast to the conventional mechanism of color formation described in previous studies. We identified the key metabolites and genes involved in the light-induced color formation of apple peel under dark conditions through metabolomics and transcriptomic analyses and explored the possible regulatory mechanism. The novel phenotypes documented in this study provide new insights into the mechanism of anthocyanin synthesis.

## Materials and methods

### Plant materials and treatments

Ten-year-old ‘Fuji’ apple trees (*M. domestica* Borkh.) on M9 rootstocks were used in this study. All trees were planted at a 1.5 × 4.0 m spacing in an orchard in Tai’an, Shandong, China (36.12°N, 117.01°E). They were approximately 4.0 m tall with a central leader, and their crop load was adjusted to 1 fruit/20 cm. The trees were maintained using conventional agricultural practices, including soil, fertilizer and water management, and disease and pest control measures. Approximately 45 days after full bloom, the fruits were bagged with double-layer paper bags (brown outside and red inside). At approximately 170 days after full bloom, fruits were removed from the trees. A total of 240 fruits were subjected to the control treatment, and the rest were placed under weak light conditions in several treatments.

Approximately 700 fruits were divided into three groups and exposed to natural light above four layers of wet gauze in an open space, three groups of apples were placed on gauze at 6:00 a.m. on the 1st, 2nd, and 3rd day respectively, which was the treatment of light induced for 3 (G3), 2 (G2), and 1 (G1) natural days. Every day light intensity were measured with a digital Lux meter (LX10108) after putting the apples, the values were 3,710.0, 3,813.3, and 3,376.7, respectively. Fruits of light induced treatment and the control group (CK) were placed in plastic turnover box before 6:00 a.m. on the 4th day, then put the turnover boxes in the cold storage without opening the compressor for dark treatment. Samples were taken at 0 (D0), 1 (D1), 3 (D3), 5 (D5), and 7 days (D7) of the dark treatment. There were five replicates of each treatment and eight fruits per replicate.

At each sampling event, the changes in peel color were determined, and the color difference of the peel was determined; the fruit peels (1 mm thick) were then removed from individual fruits using a peeler and quickly frozen in liquid nitrogen. The frozen samples were mixed in liquid nitrogen, ground into powder using a grinding machine (IKA Company, Germany), and stored at −80°C for further analysis.

### Analysis of peel color

The peel color, including the color difference and pigments, was analyzed at five stages under the three light induction treatments.

#### Color difference

The color difference of the peel was measured using a colorimeter (CR-410, Konica Minolta, Japan). The indexes included L*, a*, b*, *C*, and *h*° [*C*^2^ = (*a*^2^ + *b*^2^)/2], *h*°= tan-1(*b*/*a*) (a* > 0, b* > 0) or *h*°= tan-1(*b*/*a*) + 180 (a* < 0, b* > 0) (Wu et al., 2014).

#### Extraction and determination of chlorophyll and carotenoids

Approximately 1.0 g of peel powder was placed in 5 ml of precooled 80% acetone solution at 4°C in the dark for 24 h; the crude extract was centrifuged at 7,500 *g* for 20 min at 4°C, and the supernatant was collected. Acetone (80%) was used as the control; treatment and control samples were assayed using a spectrophotometer (UV-2450, Shimadzu, Japan) at 665 nm, 649 nm, and 470 nm. The content of chlorophyll and carotenoids was calculated using the following formula:


$$\mathrm{Ca}=13.95\,D_{665}-6.88\,D_{649}\,\left(\mathrm{mg}\cdot l^{-1}\right)$$

$$\mathrm{Cb}=24.96\,D_{649}-7.32\,D_{665}\,\left(\mathrm{mg}\cdot l^{-1}\right)$$

$$\mathrm{Cx}.c=\left(D_{470}-2.05\,\mathrm{Ca}-114.8\,\mathrm{Cb}\right)/245\,\left(\mathrm{mg}\cdot l^{-1}\right)$$

Pigment content (mg⋅g^–1^) = pigment concentration (mg⋅l^–1^) × extract volume (l) × dilution ratio/sample weight

#### Extraction and determination of anthocyanins

Anthocyanins were extracted and analyzed following a previously described method (Zhang et al., 2010). Approximately 0.5 g of crushed peel was placed in 2 ml of precooled 0.1% hydrochloric acid methanol and subjected to ultrasonic extraction for 30 min. The homogenate was centrifuged at 12,000 *g* for 10 min. After discarding the supernatant, the residue was extracted with 1 ml of extracting solution. After centrifugation, the supernatant was mixed and injected into the reaction bottle through a 0.45-μm filter, and the filtrate was the anthocyanin extract. The content of anthocyanins was determined using an Agilent 1,200 liquid chromatograph equipped with a diode array detector (Agilent Technology, Palo Alto, CA, United States); anthocyanins were separated on an Inertsil ODS-3 column (5.0 μm, 4.6 mm × 250 mm). Mobile phase A was a 1.6% formic acid aqueous solution, and mobile phase B was a 1.6% formic acid methanol solution. Gradient elution conditions were as follows: 85% A (0 min), 80% A (5.0 min), 72% A (10.0 min), 40% A (20.0 min), 0% A (35.0 min), and 85% A (50.0 min). The equilibrium time (10 min) was performed with a flow rate of 1.0 ml⋅min^–1^ and a column temperature of 40°C. Simultaneous monitoring was performed at 530 nm for cyanidin-3-O-galactoside.

#### Extraction, determination, and analysis of metabolites

Metabonomics analysis was performed in five treatment groups: control (CK), 1 day of natural light induction (G1), 3 days of natural light induction (G3), dark treatment for 3 days after natural light induction for 3 days (D3), and dark treatment for 7 days after natural light induction for 3 days (D7). Extraction and determination of metabolites were performed at Wuhan Metware Biotechnology Co., Ltd^1^. A total of 0.1 g of peel powder was extracted with 1 ml of 70% methanol solution at 4°C for 24 h. The homogenate was centrifuged at 10,000 *g* for 10 min at 4°C. The supernatant was filtered through a 0.22-μm Millipore filter, and the filtrate was the sample subjected to testing. Metabolites were detected using ultra-performance liquid chromatography (UPLC) (Shim-pack UFLC SHIMADZU CBM30A) and tandem mass spectrometry (MS/MS) (Applied Biosystems 6500 QTRAP). The column temperature was 40°C, the flow rate was 0.4 ml⋅min^–1^, and the injection volume was 2 μl. Mobile phase A was 0.04% acetic acid aqueous solution, and mobile phase B was acetonitrile containing 0.04% acetic acid. The elution gradient was carried out per the conditions listed in Supplementary Table 1. The voltage of the mass spectrometer was 5,500 V, the temperature of the electrospray ion source was 500°C, and the curtain gas was 25 psi. Qualitative analysis of substances was carried out according to the secondary spectrum information in the MEDB (metaware database). The relative content of metabolites was calculated according to the corrected mass spectrum peak area (Fraga et al., 2010). The differentially accumulated metabolites (DAMs) between different comparison groups were identified using the following criteria: |log_2_ (fold change)| ≥ 2 and *p*-value < 0.05.

### Transcriptome analysis

The samples collected for transcriptome analysis were the same as those used for the metabolomics analysis. The total RNA of apple peel was extracted using a polysaccharide polyphenol plant total RNA extraction kit (RNAprep Pure), and the library was constructed after RNA detection. Sequencing was carried out using the Illumina HiSeq platform; after quality control of the library, 150 bp paired-end reads were generated. The apple reference genome^2^ was used as the reference sequence for alignment analysis, which was conducted using HISAT2 (Kim et al., 2015). The genes were counted using Feature Counts software, and the level of gene expression was measured by FPKM (Fragments Per Kilobase of transcript per Million mapped reads). The differentially expressed genes (DEGs) between different comparison groups were analyzed using DESeq2 software (Yang et al., 2014). The Benjamin–Hochberg method was used to correct *P*-values, and the false discovery rate (FDR) was obtained. DEGs were screened using the following criteria: |log2 (fold change)| ≥ 2 or FDR < 0.05. KEGG analysis was conducted on the genes identified using BLAST software, and enriched KEGG pathways were those with *P* < 0.05 after correction.

### qRT-PCR analysis

qRT-PCR was used to verify the DEGs. Specific quantitative primers were designed using Primer 6.0 software (Supplementary Table 2). The quantitative analysis was performed using Tip Green qPCR SuperMix, and the reaction system (20 μl) contained the upstream and downstream fluorescent quantitative primers (1 μl), Tip Green qPCR SuperMix (10 μl), cDNA (1 μl), and ddH_2_O (1 μl). Gene expression was detected using real-time quantitative PCR (iCycler iQ5), and the reaction procedure is shown in Supplementary Table 3. Expression levels were determined using the 2^–△△CT^ method, and *MdACIIN* was the internal reference gene.

### Statistical analysis

SPSS 19.0 (IBM Corporation, United States) was used to determine significant differences between groups and conduct one-way ANOVA and Duncan multiple range tests (*p* < 0.05). GraphPad Prism software was used to make figures.

## Results

### Light-induced color formation of apple peel under dark conditions

**FIGURE 1:**
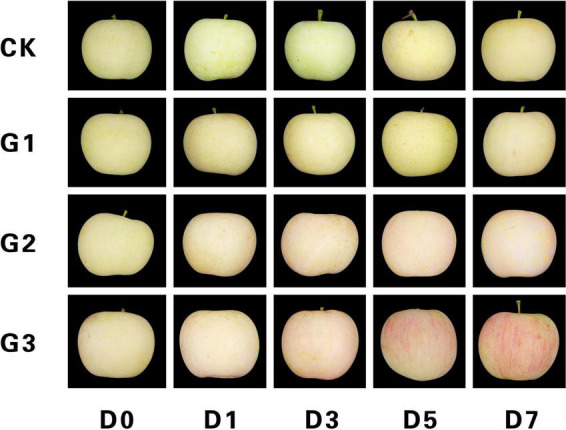
Light-induced color formation of bagged apples under dark conditions. CK, No bag removal; G1, 1 day of natural light induction; G2, 2 days of natural light induction; G3, 3 days of natural light induction; D0, 0 day of dark treatment; D1, 1 day of dark treatment; D3, 3 days of dark treatment; D5, 5 days of dark treatment; and D7, 7 days of dark treatment.

The light-induced color formation of apple peel under dark conditions is shown in Figure 1. Control and G1 peels were predominantly green at the start of the dark treatment, and yellowing occurred only in the later stages. G2 peels were light pink at D1, and red pigment deposition began to appear at D3, which was followed by a gradual deepening of the red color on the fruit surface and a uniform pink color on the peel at D7. D3 peels had a faded green color at the start of the dark treatment, and red pigment deposition increased gradually during dark treatment. A bright red stripe appeared on the peel at D5, and a bright and deep red surface similar to commercial fruits was present at D7.

### Changes in peel color difference and pigments

**FIGURE 2:**
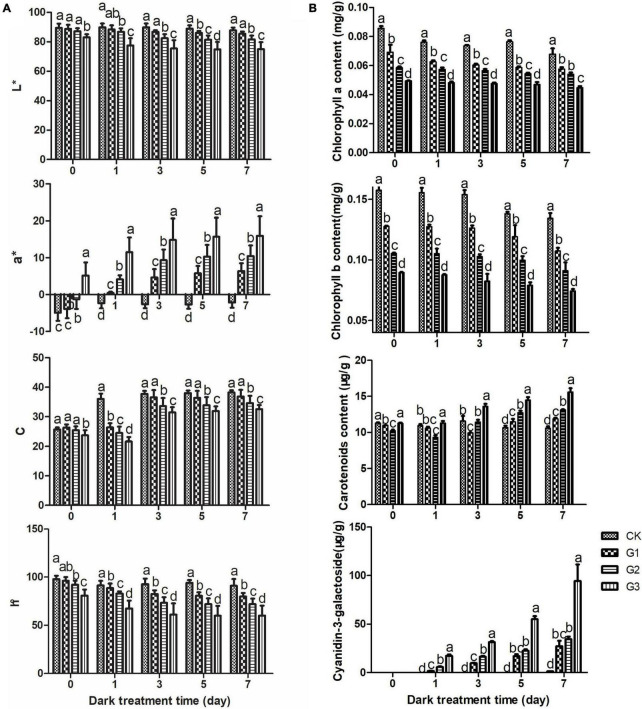
Changes in apple peel color during dark treatment. Changes in apple peel color difference values **(A)** and the pigment content **(B)** during dark treatment. L* indicates brightness. Positive a* values indicate the darkness of the red coloration and are shown in red. Negative a* values indicate the darkness of the green coloration and are shown in green. C indicates the strength of the color, and h indicates the comprehensive chroma.

Changes in the color difference of apple peel during the color formation process under dark conditions are shown in Figure 2A. The brightness L*, color intensity C, and comprehensive color h° were inversely proportional to the light induction time within each dark treatment stage, and there were significant differences among most light induction treatments. During the dark treatment, L* and h°changed little, and C increased slightly. a* significantly increased as the light induction time extended within each dark treatment stage.

The changes in pigments are shown in Figure 2B. The content of chlorophyll *a* and chlorophyll *b* decreased continuously throughout the dark treatment. The chlorophyll content decreased as the light induction time increased within each dark treatment stage, and significant differences in the chlorophyll content were observed among most treatments. The carotenoid content in the treatment group increased gradually with the dark treatment time; however, little change was observed among dark treatment stages. In the late stages of dark treatment (5 d, 7 d), the carotenoid content increased as the light induction time extended; it was highest in G3, followed by G2, G1, and CK, and significant differences were observed among treatments. The content of cyanidin-3-O-galactoside, the main red component of apple peel, increased as the dark treatment time extended and the light induction time increased. Cyanidin-3-O-galactoside was not detected in all light-induced treatments, including the 0-day dark treatment and all controls (with the exception of the 7-day dark treatment), because its content was low.

### Metabonomic analysis of light-induced color formation under dark conditions

#### Metabolite detection

**FIGURE 3:**
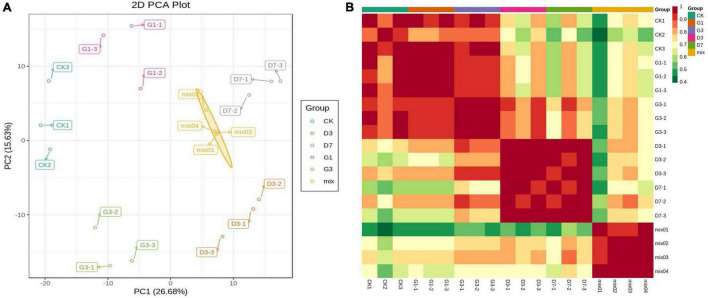
PCA plot of sample mass spectrum data **(A)** and correlation heat map of the sample **(B)**.

Principal component analysis revealed significant separation of CK, G1, G3, D3, and D7 along the first principal component and second principal component, each of which explained 26.68 and 15.63% of the variation, respectively, indicating that there were significant changes in the metabolites among the control, light induction, and dark-colored samples (Figure 3A). A correlation heat map showed that the correlation coefficients of the three biological replicates in the group and their biological repeatability were high (Figure 3B). A total of 620 metabolites were detected from five groups of samples, including 23 major categories such as anthocyanins, proanthocyanidins, amino acids and derivatives, sugars, nucleotides and derivatives, flavonoids, flavonoids, and lipids (Supplementary Table 4).

#### Identification of differentially accumulated metabolites

**FIGURE 4:**
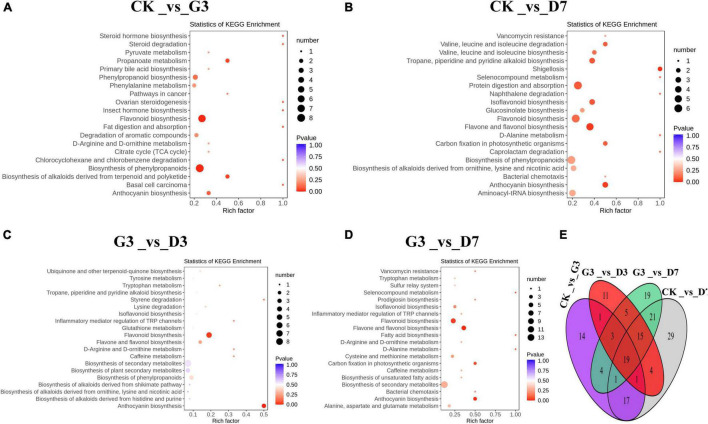
Identification and functional characterization of the differentially accumulated metabolites (DAMs) in the different treatments. KEGG enrichment analysis of the DAMs in **(A)** CK_vs_G3, **(B)** CK_vs_D7, **(C)** G3_vs_D3, and **(D)** G3_vs_D7. **(E)** Venn figure showing the shared and specific metabolites among the four groups.

Differentially accumulated metabolites (DAMs) in the four comparison groups CK_vs_G3, CK_vs_D7, G3_vs_D3, and G3_vs_D7 were analyzed (Figure 4), and there were 60, 107, 59, and 87 DAMs in each of these groups, respectively; 19 metabolites were common among these groups (Figure 4E). Among these 19 metabolites, there were 2 anthocyanins, 6 flavonoids, 6 flavonols, 2 flavanones, and 3 other substances. KEGG pathway enrichment analysis revealed that the DAMs in the four groups were significantly enriched in flavonoid biosynthesis pathway and anthocyanin biosynthesis pathway. The enrichment of the flavonoid biosynthesis pathway and anthocyanin biosynthesis pathway was extremely significant, with the exception of the CK_vs_G3 group (Figures 4A–D). This indicates that the DAMs in the flavonoids biosynthesis pathway are the key metabolites involved in the light-induced color formation of apple peel under dark conditions.

#### Analysis of flavonoids

**FIGURE 5:**
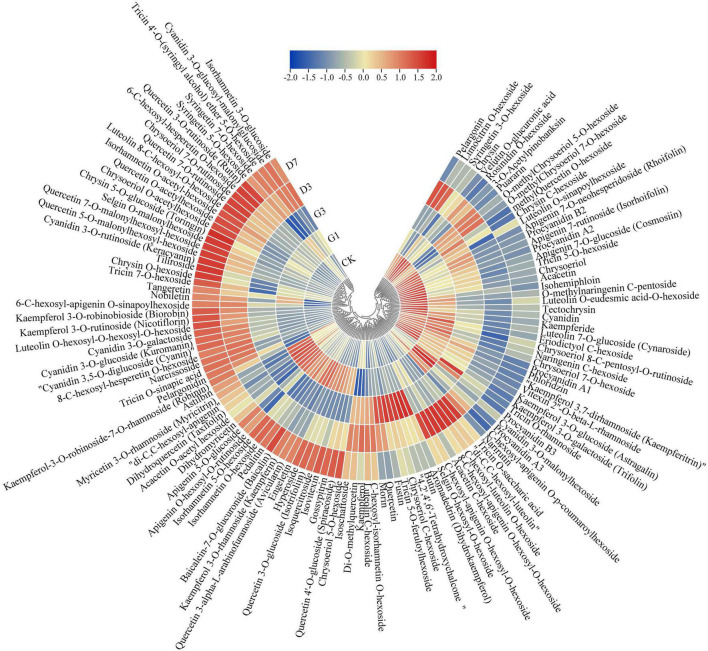
Expression of flavonoid metabolites in the different treatments. Gene expression data were normalized and plotted using Tbtools V1.075 software.

A total of 121 flavonoid metabolites were detected in the five groups of samples, including 10 anthocyanins, 5 proanthocyanidins, 62 flavonoids, 30 flavonols, and 14 flavonoids. The changes in the content of each substance among the different treatments were shown in Figure 5. Ten anthocyanins were closely related to the development of the red coloration of apple peel (Supplementary Table 5). The content of cyanidin 3-O-galactoside and cyanidin 3-O-glucoside changed substantially throughout the experimental period. These substances were not expressed in the control and G1 but were abundant in G3. They were expressed in large quantities in the dark stage, and their expression peaked at D7. These expression changes were consistent with phenotypic changes; thus, cyanidin 3-O-galactoside and cyanidin 3-O-glucoside are the key metabolites associated with the light-induced coloration of apple peel under dark conditions.

### Transcriptome analysis

#### Quality control analysis of transcriptome data

**FIGURE 6:**
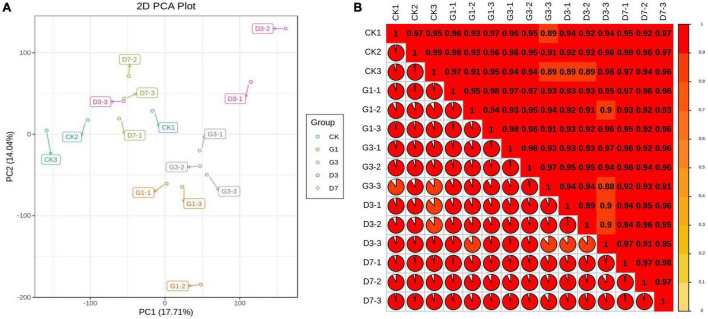
Correlation analysis between RNA-Seq replicate samples **(A)** and analysis of the principal components **(B)** of apple peel under different treatments.

The changes in the gene expression profiles of CK, G1, G3, D3, and D7 were analyzed. Three biological replications were conducted for each group. A total of 135.14 GB of clean data were obtained from the 15 peel samples; the base error rate was approximately 0.4%, and the ratios of Q20 and Q30 were 96.60 and 90.86%, respectively. This indicated that the quality of the transcriptome data of each sample was high, which suggests that our transcriptome data were accurate and reliable (Supplementary Table 6). The proportion of reads that were mapped to multiple locations in the genome was 12.49–14.23%, and the proportion of reads with map quality no less than 30 was greater than 62%, indicating that a high proportion of reads from each sample were compared against the reference genome (Supplementary Table 7). Correlation thermography showed that the square of the Pearson correlation coefficient (r^2^) of the three biological replicates in the group was greater than 0.9, indicating high reproducibility of the samples (Figure 6A). Principal component analysis revealed clustering of samples within groups and separation of samples in different groups, which indicated high repeatability of samples within groups and large differences among samples in different groups; the separation among the light treatment, dark treatment, and control was especially pronounced (Figure 6B). These findings were consistent with the metabonomics data, indicating that the accumulation of different metabolites in apple peel under dark conditions was regulated by the expression of different genes.

#### Identification of differentially expressed genes

**FIGURE 7:**
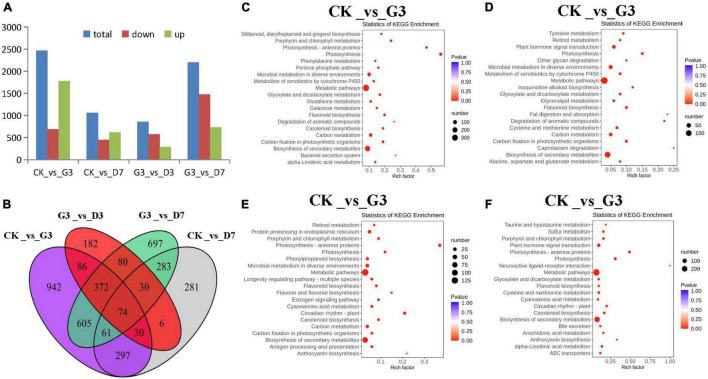
Identification and characterization of differentially accumulated metabolites (DAMs) in the peel of different treatments. **(A)** DEG statistics among the four comparison groups in the different treatments. **(B)** Venn diagram showing the shared and specific DEGs among the four comparison groups of the different treatments. KEGG enrichment analysis of the DEGs in the **(C)** CK_vs_G3, **(D)** CK_vs_D7, **(E)** G3_vs_D3, and **(F)** G3_vs_D7 groups.

Analysis of the DEGs in the four comparison groups CK_vs_G3, CK_vs_D7, G3_vs_D3, and G3_vs_D7 was conducted. A total of 2,467 DEGs were detected in the CK_vs_G3 group, including 1,781 up-regulated genes and 686 down-regulated genes. This was followed by G3_vs_D7 with 2,202 DEGs (728 up-regulated genes and 1,474 down-regulated genes). This indicated that various metabolic pathways were active in apple peel in the light induction stage; thus, there were significant differences in the DEGs between the light induction and control groups and between the light induction and dark groups (Figure 7A). A Venn diagram revealed 74 common DEGs among the four comparison groups. Among these DEGs, 34 were enriched in flavonoid metabolism pathway. A total of 27 of these were enriched in glycosylation transfer, and 16 were transcription factors such as MYB, bHLH, and NAC; 24 genes had other functions and no annotation could be recovered (Figure 7B). Analysis of KEGG pathway enrichment revealed that the DEGs were mainly enriched in metabolic pathway and secondary metabolite biosynthesis pathway, as well as the chlorophyll, carotenoid, and flavonoid metabolic pathway related to peel color, especially the anthocyanin biosynthesis pathway, and genes involved in this pathway were significantly expressed only in the groups subjected to dark treatment (Figures 7C–F). Anthocyanin synthesis is thus key to light-induced color formation under dark conditions.

#### Expression analysis of genes involved in the anthocyanin synthesis pathway

**FIGURE 8:**
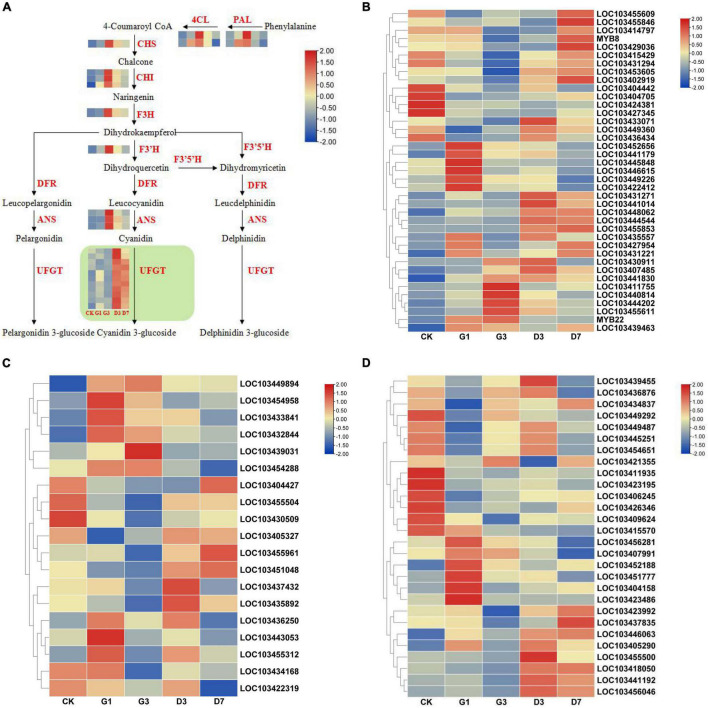
Expression analysis of the structural genes in anthocyanin synthesis pathways and transcription factors in the peel of different treatments. **(A)** Expression analysis of structural genes in the anthocyanin synthesis pathways in the peel under different treatments. Expression analysis of MYB **(B)**, bHLH **(C)**, and NAC **(D)** transcription factors.

A total of 31 structural genes annotated to eight enzymes in the anthocyanin biosynthesis pathway were identified among the DEGs (Figure 8A). The expression patterns of the photosensitive genes *MdPALs* and *MdANSs* are inconsistent with the phenotypic changes observed under dark conditions. However, some *MdUFGT* genes were highly expressed under dark conditions, including *LOC103439229*, *LOC103455827*, *LOC103443393*, *LOC103443444*, *LOC103410839*, *LOC103411018*, *LOC103438383*, *LOC103441424*, *LOC103409768*, *LOC103433538*, *LOC103411040*, and *LOC103421716*.

Transcription factors involved in anthocyanin biosynthesis mainly include MYB, bHLH, NAC, and other gene families. Ten candidate genes from the MdMYB family showing expression patterns consistent with phenotypic changes were identified, including *LOC103431271*, *LOC103441014*, *LOC103448062*, *LOC103444544*, *LOC103455853*, *LOC103435557*, *LOC103415429*, *LOC103431294*, *LOC103453605*, and *LOC103402919* (Figure 8B). Three candidate genes were identified from the MdbHLH family: *LOC103455961*, *LOC103451048*, and *LOC103435892* (Figure 8C). Four candidate genes were identified from the MdNAC family: *LOC103455500*, *LOC103418050*, *LOC103441192*, and *LOC103456046* (Figure 8D).

### qRT-PCR verification

**FIGURE 9:**
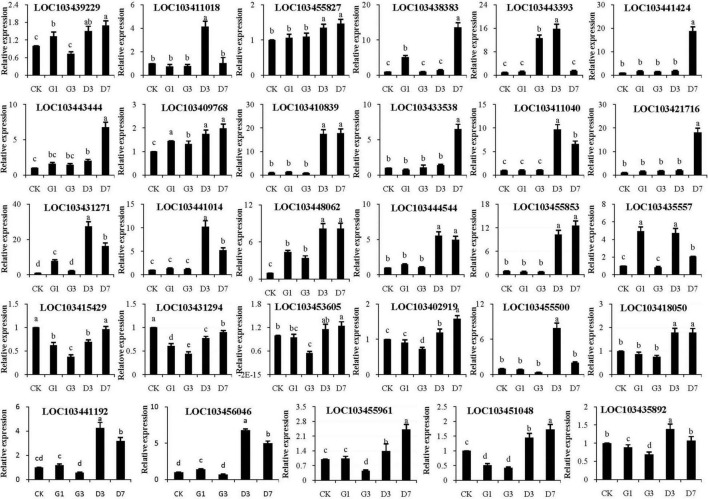
Verification of the expression of *MdUFGT* and transcription factor (*MdMYB*, *MdNAC*, and *MdbHLH*) genes in the peel in different treatments by qRT-PCR.

The selected candidate genes were verified by qRT-PCR, and the expression patterns of five *MdUFGTs* were consistent with phenotypic changes observed in experiments: *LOC103443444*, *LOC103410839*, *LOC103411018*, *LOC103441424*, and *LOC103421716* (Figure 9). Five candidate *MdMYB* genes, *LOC103431271*, *LOC103441014*, *LOC103448062*, *LOC103444544*, and *LOC10345585*, were also identified (Figure 9). Three candidate *MdNAC* genes, *LOC103455500*, *LOC103441192*, and *LOC103456046*, were identified (Figure 9). *MdbHLH* genes were not selected as candidate genes because of their small difference multiples (Figure 9). Therefore, the above candidate genes including *MdUFGTs*, *MdMYBs* and *MdNACs* may be the key to the apple’s reddening which need further research.

## Discussion

Light is an essential condition for anthocyanidin synthesis in many fruits (Maier and Hoecker, 2015). Light promotes anthocyanin accumulation by up-regulating the expression of structural genes and transcription factors involved in the anthocyanin synthesis pathway. However, the expression of these genes is significantly reduced under weak light or dark conditions, which leads to decreases in the anthocyanin content (Mol et al., 1996; Jeong et al., 2004). Previous studies have shown that anthocyanins do not accumulate or degrade in dark environments; whether color formation can occur in fruits under dark conditions following light induction has not been reported to date. We found that color formation can occur in apple peel following light induction. After light induction, anthocyanin synthesis occurred in apple peel in the dark, and color formation did not occur in apple peel without light induction (Figure 1). Fruit color is determined by chlorophyll, carotenoids, and flavonoid pigments (including anthocyanins, flavonoids, and flavonols) according to studies of apple, avocado, and lychee (Wang et al., 2002; Cox et al., 2004; Chi et al., 2021). In this experiment, the pattern of color formation of apple peel under a dark environment was similar to that observed under natural conditions. As the chlorophyll content decreased and the anthocyanin content increased in the peel, the green color of the peel faded, and the red color developed. The content of carotenoids increased, which was not consistent with expectation, possibly because the length of the dark treatment was not sufficiently long for a decline in the content of carotenoids to be detected; however, additional work is needed to verify this possibility. Analysis of color variation in the peel showed that the bright chromaticity L* and comprehensive chromaticity h° decreased, and the red/green chromaticity a* increased rapidly during the color formation process; these findings are consistent with the results of previous studies of apple (Meng et al., 2016).

The metabolic network underlying color development has been examined using metabolomics in various studies (Jian et al., 2019; Zhang et al., 2020; Zhou D. et al., 2020). In our study, 620 metabolites belonging to 23 categories were detected, including anthocyanins, proanthocyanidins, amino acids and derivatives, phenolamides, carbohydrates, flavones, flavonols, flavanones, and organic acids and derivatives. Many studies have shown that the substances associated with the development of peel color are mainly flavonoids, including flavones, flavonols, flavanones, isoflavones, and anthocyanins (Ubi et al., 2006). A total of 171 flavonoids, including 13 anthocyanins, 116 flavonoids, and 42 phenylpropanoids, were detected in the jujube varieties ‘Junzao’ and ‘Tailihong’ (Shi et al., 2020). A total of 158 flavonoids, including 13 anthocyanins, 140 flavonoids, and 5 proanthocyanidins, were detected in the jujube variety ‘Dongzao’ (Zhang et al., 2020). In our study, 121 flavonoids, including 10 anthocyanins, 5 proanthocyanidins, and 106 flavonoids, were detected. Further analysis revealed that the DAMs between the control and the different light induction and dark treatments were significantly enriched in flavonoid biosynthesis pathway and anthocyanin biosynthesis pathway. This indicates that the flavonoid biosynthesis pathway and anthocyanin biosynthesis pathway are key metabolic pathways involved in color formation in dark environments. There were 10 anthocyanins closely related to the development of the red color of apple peel, and cyanidin-3-O-galactoside and cyanidin-3-O-glucoside were the main metabolites correlated with the observed phenotypic changes. Cyanidin-3-O-galactoside accounted for 84.71% of the total pigment after 7 days of dark treatment. These findings are consistent with the results of previous studies (Treutter, 2001).

RNA-Seq is commonly used to identify DEGs and enriched metabolic pathways from large datasets, and this technique has been used in studies of pear, peach, and strawberry (Lin et al., 2018; Cao et al., 2019; Yuan et al., 2021). The expression analysis conducted in this study revealed a large number of DEGs between the light induction treatment and the control and between the light induction treatment stage and the dark treatment stage. KEGG pathway analysis revealed that the DEGs genes were enriched in metabolic pathway, biosynthesis of secondary metabolites, chlorophyll metabolism, carotenoid biosynthesis, anthocyanin biosynthesis, and other pathways, and these DEGs, especially those involved in anthocyanin biosynthesis, were significantly expressed exclusively in the dark treatment stage. This indicated that anthocyanin synthesis was the key to color formation under dark conditions.

Many studies have shown that there is a positive correlation between anthocyanin accumulation in the pericarp and one or more structural genes in the anthocyanin synthesis pathway (Takos et al., 2006; Wei et al., 2011; Zhang et al., 2020). In our study, the expression of 12 *MdUFGT* genes was low in the control and light induction stages and higher in the dark treatment stage, which was consistent with observed phenotypic changes, and the expression of other structural genes was not consistent with phenotypic changes. Therefore, *MdUFGT* is considered a key structural gene involved in the color formation of apple peel in dark environments after light induction. Transcription factors are the second largest group of regulatory proteins involved in anthocyanin synthesis, and they regulate anthocyanin synthesis by activating or inhibiting the activity of the upstream promoters of structural genes (Kobayashi et al., 2001; An et al., 2015; Jiang et al., 2021). Five MYB transcription factors and three NAC transcription factors were identified by phenotypic screening and qRT-PCR. High expression of *MdMYB*s, *MdNAC*s, and *MdUFGT*s is important for anthocyanin accumulation under dark conditions. Light-induced color formation of apple peel under dark conditions is controlled by the MdMYB and MdNAC transcription factors, which act on the promoters of *MdUFGT*s to regulate anthocyanin synthesis. This mechanism requires verification by yeast single-hybrid assays, electrophoretic mobility shift assays, and transient transformation tests.

## Conclusion

Under dark conditions, anthocyanins could be synthesized in apple peel following light induction, and this was associated with a decrease in bright chromaticity L*, comprehensive chromaticity h°, and chlorophyll content and an increase in red/green chromaticity a* and the anthocyanin content. Metabolic analysis indicated that cyanidin-3-O-galactoside and cyanidin-3-O-glucoside were key metabolites. Transcriptome analysis revealed that *MdUFGT*s, *MdMYB*s, and *MdNAC*s were the key transcription factors involved in this color formation process. Further study of the roles of the 13 candidate genes identified by qRT-PCR could provide new insight into the light-induced color formation of apple peel under dark conditions.

## Data availability statement

The original contributions presented in the study are publicly available. This data can be found here: NCBI GSE118455 and MetaboLights MTBLS4989.

## Author contributions

XX, XZ, and JW designed the experiment. XX, RC, and XH performed the experiment and analyzed the data. XX and ST wrote the manuscript. All authors have read and approved the final version of the manuscript.

## Footnotes

1
https://www.metware.cn

2
https://www.rosaceae.org/
