# Supplementary material for: Clarifying the mechanisms of the light-induced color formation of apple peel under dark conditions through metabolomics and transcriptomic analyses
Source: Front Plant Sci. 2022 Jul 28;13:946115. doi: 10.3389/fpls.2022.946115 (PMC9366354; doi:10.3389/fpls.2022.946115)
Supplement: Supplementary file 1 [file Table_1.DOCX]

Table S1 Elution gradient in HPLC

| Time (min) | Mobile phase (v/v) |
| --- | --- |
| 0 | 95:5 |
| 11.0 | 5:95 |
| 12.0 | 5:95 |
| 12.1 | 95:5 |
| 15.0 | 95:5 |
