# Supplementary material for: Clarifying the mechanisms of the light-induced color formation of apple peel under dark conditions through metabolomics and transcriptomic analyses
Source: Front Plant Sci. 2022 Jul 28;13:946115. doi: 10.3389/fpls.2022.946115 (PMC9366354; doi:10.3389/fpls.2022.946115)
Supplement: Supplementary file 2 [file Table_2.DOCX]

Table S2 Quantitaive primers of qRT-PCR

| Gene ID | Gene name | | Primer sequences (5′-3′) |
| --- | --- | --- | --- |
|  | | Actin | F:TGACCGAATGAGCAAGGAAATTACT |
|  | |  | R:TACTCAGCTTTGGCAATCCACATC |
| *LOC103455961* | | bHLH | F:CTTTGTGTATTTCTCTCCTTCGCT |
|  | |  | R:TTGTTATGTGGGTTTTGGTCTGTA |
| *LOC103451048* | | bHLH | F:GACATGCAGTAAAAGAACGGACAC |
|  | |  | R:GAAAAAACCCAGCAAAGAAAAGAC |
| *LOC103435892* | | bHLH | F:CCTTGCTGAAAGGGTAAGAAGAGA |
|  | |  | R:GTCCACAGCCCCATTAGAAAGTA |
| *LOC103455500* | | NAC | F:CAACCTGAGAGACCGAAAATATCC |
|  | |  | R:CATCCCAACCAGCACACCTACA |
| *LOC103418050* | | NAC | F:AGCCAAAAACTCCAAGTCTCACC |
|  | |  | R:GCTCTCCTTCTTTCTCCTCCTCA |
| *LOC103441192* | | NAC | F:TCACCCTCTCTCTCTCCCTCTCT |
|  | |  | R:AAGTACCACTCCTTTTCTCCCATG |
| *LOC103456046* | | NAC | F:GAGTTTGCCCCCAATTACTTTTC |
|  | |  | R:CTCCTTTTCTCCCATTTTCGC |
| *LOC103431271* | | MYB | F:CTGATTGCCATTCTTCATTTGTG |
|  | |  | R:GCTCCTCTGTTGAGTCCTTCCTT |
| *LOC103441014* | | MYB | F:TGGTAGCAATAGGGGGAGGTG |
|  | |  | R:ACTTGAGAAGTTTCTTTCGAGGATC |
| *LOC103448062* | | MYB | F:GAGATGTGGGAAAAGCTGCAGA |
|  | |  | R:GGAAAAGAGAAACAGAGTTGGTG |
| *LOC103444544* | | MYB | F:TATCATCATTCGCTTTGTCATTGG |
|  | |  | R:TGTAGAGGTGCTGTTTCTTTCGGT |
| *LOC103455853* | | MYB | F:GGGTATCATCATTCGCTTTGTC |
|  | |  | R:GTAGAGGTGCTGTTTCTTTCGG |
| *LOC103435557* | | MYB | F:AGCAGGTTTGCTGAGATGTGGT |
|  | |  | R:AGAGGATGGTAAATTTTGGCGT |
| *LOC103415429* | | MYB | F:CGCCATAAAGAACCACTGGAAC |
|  | |  | R:GACCTGAGACGAGGGAGAGACA |
| *LOC103431294* | | MYB | F:CGTCTCTTTTTTTCAACCTGGG |
|  | |  | R:CTGTAACGCTTCGTCTTCCTCG |
| *LOC103453605* | | MYB | F:CCCAAAATAGCATCCTCTCTGC |
|  | |  | R:TCAACTCCCTTCACTTTCCGTC |
| *LOC103402919* | | MYB | F:CCAAGCACCAATCCTTCACAAC |
|  | |  | R:GAATGACACACCAAACACACAAAAAG |
| *LOC103439229* | | UFGT | F:GATTTGGTGAAACAGGAAGGGG |
|  | |  | R:CATGTCGGAGGAGTGGAGGACT |
| *LOC103455827* | | UFGT | F:GTAAATGAAAAAAAACCCCAAAAAG |
|  | |  | R:AGAGGGAGACGAGTGAAAAGACG |
| *LOC103443393* | | UFGT | F:CGCCACTTTCGCCTCAATCTA |
|  | |  | R:GCCCCTGCCCAGTAATCCTAT |
| *LOC103443444* | | UFGT | F:ACCTTCTCTTCCACTTCCCACA |
|  | |  | R:TCGTTCCCAGTTCAGATCATTC |
| *LOC103410839* | | UFGT | F:CTCAATCAAATACCAGAACACCCC |
|  | |  | R:ACTGCCAAAATTCACATACACAAC |
| *LOC103411018* | | UFGT | F:CAGGGAGAAGGAGAAATGGG |
|  | |  | R:GTAAAGTGCGTGACGAACGG |
| *LOC103438383* | | UFGT | F:TCCACCCTAATAACCACCCCTC |
|  | |  | R:GCTTCTTGTCAGCTTGATCTCAAC |
| *LOC103441424* | | UFGT | F:CTCAATCAAATACCAGAACACCCC |
|  | |  | R:ATACTGCCAAAATTCACATACACAAC |
| *LOC103409768* | | UFGT | F:CCCCACATACTACTTCTGCCCT |
|  | |  | R:CATTTCCCGATTCTTTTTCATCG |
| *LOC103433538* | | UFGT | F:GTTTTGAGCACGTTACGCAGC |
|  | |  | R:CCACCACTATCACTTTTCCATTG |
| *LOC103411040* | | UFGT | F:CCCTTGCTGATGCTTCTGGT |
|  | |  | R:CGATTGACATTGCCGTCTTG |
| *LOC103421716* | | UFGT | F:AACAGATTTTGGAGTTTGGTTGG |
|  | |  | R:GTTTAAGATTTGCTCTTGAGGGC |
