# Supplementary material for: Clarifying the mechanisms of the light-induced color formation of apple peel under dark conditions through metabolomics and transcriptomic analyses
Source: Front Plant Sci. 2022 Jul 28;13:946115. doi: 10.3389/fpls.2022.946115 (PMC9366354; doi:10.3389/fpls.2022.946115)
Supplement: Supplementary file 3 [file Table_3.DOCX]

Table S3 qRT-PCR reaction procedure

| Temperature (action) | Time |
| --- | --- |
| 94℃（Pre-denaturation） | 30 s |
| 94℃(denaturation) | 5 s |
| 40–45 cycles 50–60℃（annealing） | 15 s |
| 72℃（extend） | 10 s |
| 72℃（extend） | 5 min |
