# Supplementary material for: Clarifying the mechanisms of the light-induced color formation of apple peel under dark conditions through metabolomics and transcriptomic analyses
Source: Front Plant Sci. 2022 Jul 28;13:946115. doi: 10.3389/fpls.2022.946115 (PMC9366354; doi:10.3389/fpls.2022.946115)
Supplement: Supplementary file 4 [file Table_4.DOCX]

| Serial number | Class | Quantity |
| --- | --- | --- |
| 1 | Anthocyainin | 10 |
| 2 | Proanthocyanidins | 5 |
| 3 | Amino acid and derivatives | 77 |
| 4 | Phenylpropanoids | 44 |
| 5 | Alcohols | 18 |
| 6 | Polyphenol | 12 |
| 7 | Phenolamides | 9 |
| 8 | Nucleotide and derivates | 46 |
| 9 | Flavone | 62 |
| 10 | Flavonol | 30 |
| 11 | Flavonoid | 14 |
| 12 | Flavanone | 13 |
| 13 | Quinones | 2 |
| 14 | Alkaloids | 22 |
| 15 | Carbohydrates | 21 |
| 16 | Terpene | 17 |
| 17 | Vitamins and derivatives | 16 |
| 18 | Isoflavone | 6 |
| 19 | Indole derivatives | 3 |
| 20 | Organic acids and derivatives | 94 |
| 21 | Sterides | 5 |
| 22 | Lipids | 65 |
| 23 | Others | 29 |
| Totle |  | 620 |

Table S4 Type and quantity of metabolites
