# Supplementary material for: Clarifying the mechanisms of the light-induced color formation of apple peel under dark conditions through metabolomics and transcriptomic analyses
Source: Front Plant Sci. 2022 Jul 28;13:946115. doi: 10.3389/fpls.2022.946115 (PMC9366354; doi:10.3389/fpls.2022.946115)
Supplement: Supplementary file 5 [file Table_5.DOCX]

Table S5 Relative expression of anthocyanins in apple peel under different treatments

| Anthocyanin | CK | G1 | G3 | D3 | D7 |
| --- | --- | --- | --- | --- | --- |
| Cyanidin 3-O-glucosyl-malonylglucoside | 7.90E+04 | 1.11E+05 | 7.77E+04 | 1.66E+05 | 1.70E+05 |
| Cyanidin 3-O-malonylhexoside | 8.77E+04 | 2.37E+05 | 5.23E+04 | 3.71E+04 | 6.91E+04 |
| Rosinidin O-hexoside | 4.49E+05 | 3.79E+05 | 3.51E+05 | 4.23E+05 | 3.15E+05 |
| Cyanidin 3-O-glucoside | 0.00 | 0.00 | 4.11E+06 | 1.87E+07 | 2.09E+07 |
| Pelargonidin | 1.81E+05 | 1.61E+05 | 2.17E+05 | 2.43E+05 | 2.51E+05 |
| Cyanidin 3-O-rutinoside | 1.90E+05 | 1.96E+05 | 2.53E+05 | 5.79E+05 | 1.03E+06 |
| Cyanidin 3,5-O-diglucoside | 0.00 | 0.00 | 1.92E+06 | 6.34E+06 | 7.30E+06 |
| Pelargonin | 1.45E+06 | 1.33E+06 | 1.17E+06 | 1.64E+06 | 1.10E+06 |
| Cyanidin | 1.51E+05 | 1.16E+05 | 1.04E+05 | 8.26E+04 | 9.67E+04 |
| Cyanidin 3-O-galactoside | 0.00 | 0.00 | 3.43E+07 | 1.45E+08 | 1.73E+08 |
