# Supplementary material for: Clarifying the mechanisms of the light-induced color formation of apple peel under dark conditions through metabolomics and transcriptomic analyses
Source: Front Plant Sci. 2022 Jul 28;13:946115. doi: 10.3389/fpls.2022.946115 (PMC9366354; doi:10.3389/fpls.2022.946115)
Supplement: Supplementary file 6 [file Table_6.DOCX]

Table S6 Quality control data of transcriptome sequencing

| Sample | Clean reads | Clean bases | Error rate | Q20 | Q30 | GC content |
| --- | --- | --- | --- | --- | --- | --- |
| CK-1 | 55,511,768 | 8,326,765,200 | 0.400 | 96.615 | 90.890 | 47.380 |
| CK-2 | 57,569,380 | 8,635,407,000 | 0.385 | 96.760 | 91.205 | 47.655 |
| CK-3 | 55,229,142 | 8,284,371,300 | 0.405 | 96.545 | 90.745 | 47.530 |
| G1-1 | 56,400,336 | 8,460,050,400 | 0.400 | 96.615 | 90.865 | 47.355 |
| G1-2 | 61,668,766 | 9,250,314,900 | 0.400 | 96.560 | 90.770 | 47.140 |
| G1-3 | 62,324,504 | 9,348,675,600 | 0.415 | 96.390 | 90.415 | 47.750 |
| G3-1 | 54,041,866 | 8,106,279,900 | 0.385 | 96.785 | 91.230 | 47.380 |
| G3-2 | 61,297,726 | 9,194,658,900 | 0.415 | 96.435 | 90.525 | 47.360 |
| G3-3 | 55,904,386 | 8,385,657,900 | 0.375 | 96.865 | 91.420 | 47.310 |
| D3-1 | 63,573,380 | 9,536,007,000 | 0.375 | 96.845 | 91.415 | 47.345 |
| D3-2 | 61,540,484 | 9,231,072,600 | 0.395 | 96.640 | 90.975 | 47.420 |
| D3-3 | 58,640,268 | 8,796,040,200 | 0.385 | 96.725 | 91.140 | 47.530 |
| D7-1 | 60,392,530 | 9,058,879,500 | 0.415 | 96.405 | 90.505 | 47.455 |
| D7-2 | 66,469,646 | 9,970,446,900 | 0.440 | 96.130 | 89.955 | 47.565 |
| D7-3 | 70,305,542 | 10,545,831,300 | 0.400 | 96.610 | 90.900 | 47.550 |

Q20, the percentage ratio of the base number and total base number with Qphred no less than 20; Q30, the percentage ratio of the base number and total base number with Qphred no less than 30; GC content, the percentage ratio of the G and C content and the total base number in clean reads.
