# Supplementary material for: Clarifying the mechanisms of the light-induced color formation of apple peel under dark conditions through metabolomics and transcriptomic analyses
Source: Front Plant Sci. 2022 Jul 28;13:946115. doi: 10.3389/fpls.2022.946115 (PMC9366354; doi:10.3389/fpls.2022.946115)
Supplement: Supplementary file 7 [file Table_7.DOCX]

Table S7 Reads and reference genome mapping data

| Sample | Total reads | multiple mapped reads（%） | Unmapped reads（%） | mapQuality>= 30（%） | Non-splice reads（%） | Splice reads（%） |
| --- | --- | --- | --- | --- | --- | --- |
| CK-1 | 64,171,185 | 13.49 | 13.08 | 62.98 | 37.57 | 25.42 |
| CK-2 | 66,801,288 | 13.82 | 12.88 | 62.79 | 37.07 | 25.72 |
| CK-3 | 64,095,282 | 13.83 | 13.13 | 62.72 | 37.22 | 25.50 |
| G1-1 | 64,452,834 | 12.49 | 13.53 | 64.04 | 38.02 | 26.03 |
| G1-2 | 71,799,270 | 14.11 | 12.68 | 62.55 | 37.60 | 24.96 |
| G1-3 | 76,665,528 | 18.71 | 12.17 | 57.30 | 34.44 | 22.85 |
| G3-1 | 62,296,727 | 13.25 | 13.16 | 63.32 | 37.34 | 25.98 |
| G3-2 | 71,034,919 | 13.71 | 13.34 | 62.48 | 36.92 | 25.56 |
| G3-3 | 64,542,980 | 13.38 | 12.21 | 63.72 | 37.69 | 26.04 |
| D3-1 | 73,224,733 | 13.18 | 12.96 | 63.38 | 37.78 | 25.60 |
| D3-2 | 70,812,539 | 13.09 | 13.54 | 63.05 | 37.39 | 25.66 |
| D3-3 | 68,372,271 | 14.23 | 13.13 | 62.29 | 36.82 | 25.46 |
| D7-1 | 69,569,239 | 13.19 | 13.35 | 63.24 | 36.79 | 26.45 |
| D7-2 | 76,604,339 | 13.23 | 13.69 | 62.94 | 36.34 | 26.60 |
| D7-3 | 81,387,157 | 13.62 | 12.87 | 62.97 | 36.65 | 26.33 |

Multiple mapped reads, the number of reads aligned to multiple locations in the genome; unmapped reads, the number of reads not aligned in the genome; map Quality>= 30, the number of reads with alignment quality no less than 30; non-splice reads, the number of reads aligned in the genome without splitting; and splice reads, the number of reads aligned to multiple positions in the genome.
